# Human immunodeficiency virus infection in breast cancer patients: The prevalence thereof and its effect on breast cancer characteristics at Dr. George Mukhari Academic Hospital Breast Clinic, Ga-Rankuwa, South Africa

**DOI:** 10.4102/sajr.v22i2.1361

**Published:** 2018-08-30

**Authors:** Nikoli van Zyl, Cornelia Minné, Dikeledi H. Mokone

**Affiliations:** 1 Department of Diagnostic Radiology, Dr. George Mukhari Academic Hospital, South Africa; 2 Department of Diagnostic Imaging, Dr. George Mukhari Academic Hospital, South Africa; 3 Department of Surgery, Dr. George Mukhari Academic Hospital, South Africa

## Abstract

**Background:**

Since the advent of highly active anti-retroviral therapy, improved immune functioning and prolonged survival of Human immunodeficiency virus (HIV)-positive patients has been accompanied by an increased incidence of non-AIDS-defining cancers (NADC). Breast cancer is the most prevalent NADC among HIV-positive women. However, data regarding the interaction between these two diagnoses remain limited.

**Objectives:**

To determine the effect of HIV status on the presentation of breast cancer patients at Dr. George Mukhari Academic Hospital (DGMAH).

**Methods:**

The age, gender, HIV status, CD4 count and tumour node metastases stage at presentation were recorded from the files of patients with histologically proven breast carcinoma, who had presented to the breast clinic at DGMAH from 01 January 2013 to 30 November 2017. Histological subtypes and molecular markers were retrieved from the National Health Laboratory Service. Prevalence of HIV among breast cancer patients was calculated. Cross-tabulations compared the variables between HIV-positive and HIV-negative groups. Statistical significance was assessed using Fisher’s Exact Test.

**Results:**

HIV status was determined in 129 breast cancer patients. Eighty (62.02%) were HIV-negative and 49 (37.98%) were HIV-positive. All patients were female. The mean age at presentation with breast cancer in the HIV-positive group was approximately 10 years younger, compared to the entire population and to the HIV-negative group (*p* < 0.0001). No further statistically significant associations were observed concerning HIV status and other variables.

**Conclusion:**

HIV-positive women present with breast cancer at a significantly younger mean age. Breast cancer screening protocols may need to be adjusted accordingly in such patients.

## Introduction

Human immunodeficiency virus (HIV) is a major burden in sub-Saharan Africa, with up to 70% of global HIV cases being reported in this region.^1^ In 2017, an estimated 12.6% (10.3% in 2002) of the total South African population were infected with HIV. According to mid-year population estimates for 2017 released by Statistics South Africa, an estimated 17.98% of South African adults (ages 15–49) were HIV-positive, and of these, 21.2% were women aged 15–49.^2,3^

Recent studies have indicated that, since the advent of highly active anti-retroviral therapy (HAART), there has been an increase in the average life expectancy of HIV-positive patients from 49.2 years in 2003 to 60.5 years in 2011.^4^ Since the implementation of the National Anti-retroviral (ARV) Programme in 2005, the documented number of acquired immunodeficiency syndrome (AIDS)-related deaths has decreased by over 60% between 2006 and 2017.^3^

Improvements in immune function because of HAART has also led to a decrease in the incidence of AIDS-defining malignancies, such as cervical cancer, non-Hodgkin’s lymphoma and Kaposi’s sarcoma. Conversely, the incidence of non-AIDS-defining cancers (NADC) has increased among HIV-positive patients.^4,5^ This is partially due to an increase in life expectancy among HIV-positive patients; however, infection with human papillomavirus (HPV) and Hepatitis B and C, as well as high-risk behaviours such as smoking also play a causative role.^5,6^

Breast cancer is the most prevalent type of NADC among HIV-positive women.^7^ Worldwide, it is the most commonly diagnosed malignancy among women, regardless of immune status and socioeconomic standing.^8,9^ Breast cancer accounts for nearly a quarter of cancer diagnoses, and up to 14% of cancer-related deaths globally.^10^

Mid- to high-income regions generally exhibit a much higher incidence of breast cancer than low-income regions. Although sub-Saharan Africa presents one of the lowest incidences of breast cancer worldwide, there has been an alarming rise in recent years. This is postulated to be because of increased awareness and screening, as well as changes in reproductive patterns and lifestyle.^7,9,10^ Higher mortality rates in countries with historically lower incidence rates are thought to be because of late detection and limited access to treatment.^7^

Data regarding breast cancer on the African continent remains scarce because of poor record-keeping and data management.^7,11^ The National Cancer Registry in South Africa (SA) is merely a pathology-based registry, which underestimates the incidence of cancer and is not updated regularly.^12^

Three recently published literature reviews focussing on breast cancer (not including HIV) in Africa, sub-Saharan Africa and SA reported that patients presented at a younger age and at a more advanced stage than patients from higher-income countries.^7,11,13^ The average age at presentation in women in West Africa, Kenya and Tanzania ranges from 35 to 45 years.^11^ In SA, the median age at presentation with breast cancer is 54–56 years,^14,15,16^ versus 61 years in the United States of America (US).^17^

In SA, 50%–55% of patients were diagnosed with an advanced stage of breast cancer.^6,11,14^ In the rest of Africa, the presence of advanced-stage disease varied from 33% in Morocco to 72.8% in Nigeria and 89.6% in Kenya.^11^ These figures are evidently much higher than the 8.4% observed in the US.^17^

Both local and international studies proved that patients living with HIV tend to present with breast cancer at a younger age relative to HIV-negative patients.^6,18^ No relation could be established between HIV and stage at diagnosis, tumour grade, tumour markers or molecular subtype. However, data was not conclusive, and further research was proposed.^6,7,18,19^ Literature reviews regarding the treatment of breast cancer in Africa, sub-Saharan Africa and abroad have reported that age and stage at presentation of breast cancer is not influenced by the CD4 count of HIV-positive patients.^7,11,18^

Several global studies have investigated the existence of a relationship between HIV and breast cancer, and whether HIV is promotive or preventative to breast cancer.^7,20,21^ The proposed protective effect of HIV on breast cancer is explained by the binding of the main HIV coreceptor (CXCR4), which is expressed inter alia on hyperplastic and malignant breast duct cells. Although the exact mechanism remains unknown (it is suggested that activation of cell death pathways are involved),^7^ HIV CXCR4 tropism is associated with a lower risk of developing breast cancer.^20,21^ Conversely, it is also postulated that tumour progression via immune signalling, neo-angiogenesis and the metastatic spread of breast cancer may be induced by concomitant infection with HIV, which is attributed to 17 genes found in both diseases.^7^

If the hypothesis that HIV influences the presentation of breast cancer proves to be correct, a modified screening program for breast cancer could be formulated, which targets patients at a younger age if they are HIV-positive. Early diagnosis and treatment with improved outcomes are then possible.

## Methods

This study is a descriptive study with retrospective enrolment and received ethical clearance from the Sefako Makgatho University Research Ethics Committee (SMUREC/M/11/2016:PG).

The present study included the files of all patients who had presented to the breast clinic at Dr. George Mukhari Academic Hospital (DGMAH) from 01 January 2013 to 30 November 2017 with histologically proven breast carcinoma and a known HIV status. No HIV testing was performed for the purpose of the present study. The HIV statuses were obtained from patient files, the National Health Laboratory Service (NHLS) or by patient reporting.

The age, gender, HIV status, CD4 counts in cells per microliter (µL) and tumour node metastases (TNM) stage at presentation were recorded. Histological subtypes and tumour molecular subtypes were retrieved from the NHLS and incorporated in the data.

Demographic details were descriptively summarised using frequency tables and graphs. Mean and standard deviation (minimum and maximum) was calculated for age at presentation. The prevalence of HIV in breast cancer patients was also calculated, and subsequent cross-tabulations were constructed between HIV status and age at presentation, stage of disease at presentation, histological subtypes and molecular subtypes.

We also compared the stages of presentation and CD4 count at presentation. For the purposes of the present study, patients were divided into four groups according to their CD4 count: CD4 counts of less than 200 cells/µL (thus AIDS clinically), 201 cells/µL – 500 cells/µL, 501 cells/µL – 1000 cells/µL and over 1000 cells/µL. The stage of breast cancer at presentation was also recorded for each group.

Fisher’s Exact Test was performed for the aforementioned comparisons to assess statistical significance. All statistical analyses were performed using the SAS programme (SAS Institute Inc., Carey, NC, US, Release 9.4), running on Microsoft Windows.

## Results

**FIGURE 1:**
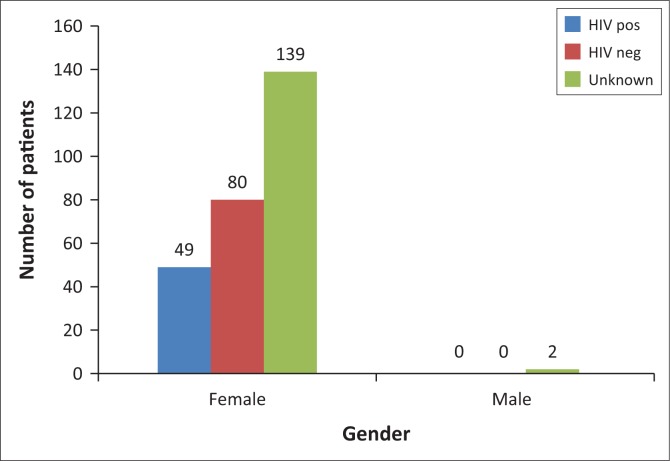
Depiction of human immunodeficiency virus status according to gender.

A total of 270 patients who had received a positive breast cancer diagnosis between 01 January 2013 and 30 November 2017 possessed files adequate for enrolment in the present study; 268 females and 2 males were observed. None of the male patients had a known HIV status and thus were not considered for further evaluation (Figure 1).

Of the 270 patients diagnosed with confirmed breast cancer, 129 (49% of 270) patients had a known HIV status. Of these, 80 (62.02% of 129) patients were HIV-negative and 49 (37.98% of 129) were HIV-positive. All patients were females.

**FIGURE 2:**
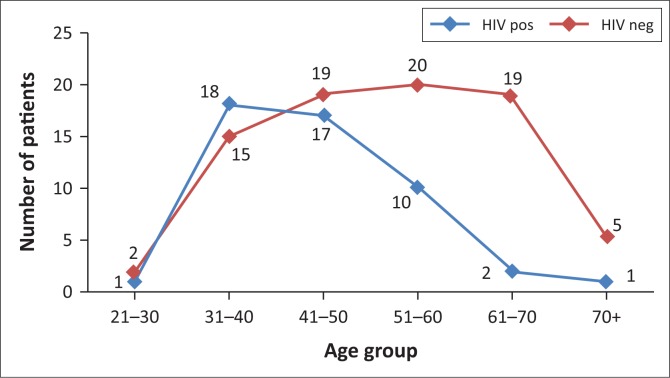
Summary of the age distribution of human immunodeficiency virus status in breast cancer patients.

**FIGURE 3:**
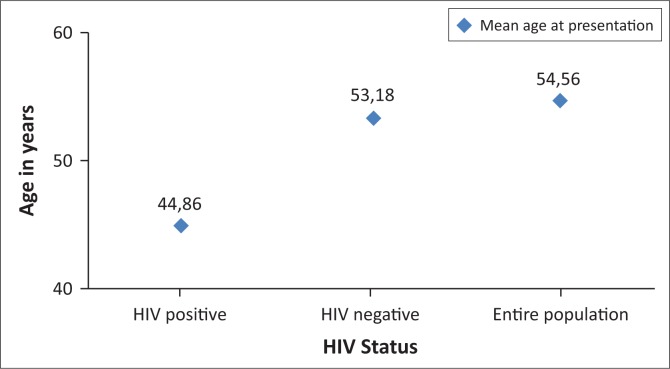
Mean age at presentation.

The mean age at presentation in HIV-negative patients was 53.18 years (±12.48), differing only slightly from that of the entire population, which was 54.56 years (±13.62) (Figure 2). However, the patients in the HIV-positive group exhibited a much younger mean age at presentation of 44.86 years (±9.00) (*p* < 0.0001) – nearly 10 years younger than their HIV-negative peers (Figure 3).

**FIGURE 4:**
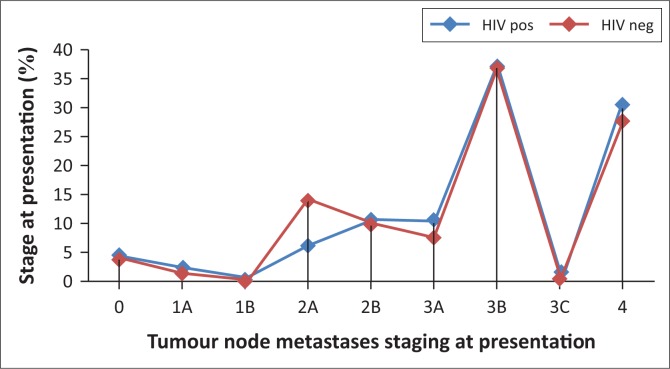
Summary of stage at presentation and human immunodeficiency virus status.

Despite a significant difference in age at presentation, the stage at presentation did not vary significantly between the HIV-positive and HIV-negative groups (*p* = 0.8912). A slightly higher percentage (67.34%) of HIV-positive patients presented with advanced stages of breast cancer (stages 3B and 4) when compared to HIV-negative patients (63.75%) (Figure 4).

Analysis of CD4 counts among HIV-positive patients revealed that 10 (25.64%) had a CD4 count of 0 cells/µL – 200 cells/µL, 16 (41.03%) had a CD4 count of 201 cells/µL – 500 cells/µL, 12 (30.77%) had a CD4 count of 501 cells/µL – 1000 cells/µL and one (2.56%) had a CD4 count of over 1000 cells/µL at presentation. CD4 counts were unavailable for 10 (25.64%) patients.

**FIGURE 5:**
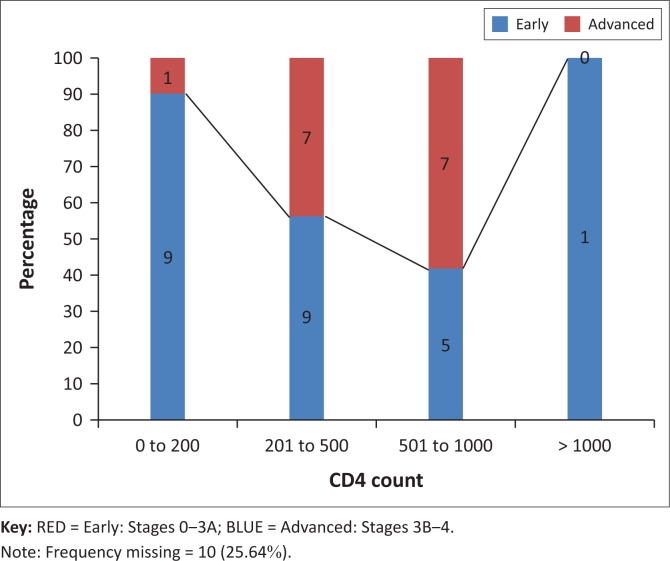
Ratio of patients presenting with early vs. advanced stage disease according to the CD4 count.

Notably, among those with known CD4 counts, the percentage of patients who presented with advanced stages of breast cancer increased with declining CD4 counts (Figure 5). Overall, 90% of patients with a CD4 count of 0 cells/µL – 200 cells/µL, 56% with 201 cells/µL – 500 cells/µL and 42% with 500 cells/µL – 1000 cells/µL exhibited advanced stages of breast cancer at the time of first staging, thereby demonstrating an inverse relationship. Only one HIV-positive patient exhibited a CD4 count over 1000 cells/µL, and presented with stage 4 breast cancer, which did not follow the trend demonstrated in other groups (Figure 5).

**FIGURE 6:**
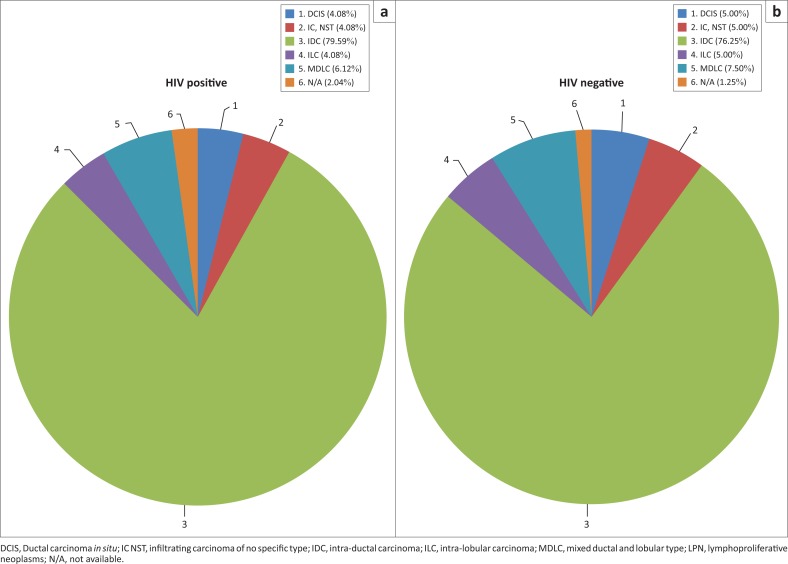
Percentages of different histological subtypes in relation to human immunodeficiency virus status: (a) HIV-positive and (b) HIV-negative.

As expected, no significant difference (*p* = 0.9380) in the histological subtype of breast cancer was present upon comparing HIV-positive to HIV-negative patients (Figure 6). The histopathology report for one HIV-negative patient could not be traced, as the biopsy was performed in private practice and was not included in the file (1.25% of 80). The patient was referred to our institution with confirmed stage 4 breast carcinoma.

A number of specimens (indicated in the individual tables) were insufficient for the performance of some or all of the receptor status testing. The status of oestrogen, progesterone, human epidermal growth factor and KI-67 protein receptors in the HIV-positive and HIV-negative patients are presented in Tables 1, 2, 3 and 4, respectively.

**TABLE 1:** Oestrogen receptor status by human immunodeficiency virus status (*p* = 0.8336).

Oestrogen	HIV				Total
	Neg		Pos		
	*n*	%	*n*	%	
Neg	23	35.38	13	31.71	36
Pos	42	64.62	28	68.29	70

**Total**	**65**	**100**	**41**	**100**	**106**

Note: Frequency missing (*n* = 23).

HIV, human immunodeficiency virus; Neg, negative; Pos, positive.

**TABLE 2:** Progesterone receptor status by human immunodeficiency virus status (*p* = 1.00).

Progesterone	HIV				Total
	Neg		Pos		
	*n*	%	*n*	%	
Neg	31	47.69	20	48.78	51
Pos	34	52.31	21	51.22	55

**Total**	**65**	**100**	**41**	**100**	**106**

Note: Frequency missing (*n* = 23).

HIV, human immunodeficiency virus; Neg, negative; Pos, positive.

**TABLE 3:** Human epidermal growth factor receptor 2 status by human immunodeficiency virus status (*p* = 0.5157).

HER-2	HIV				Total
	Neg		Pos		
	*n*	%	*n*	%	
Neg	46	71.88	26	65	72
Pos	18	28.13	14	35	32

**Total**	**64**	**100**	**40**	**100**	**104**

Note: Frequency missing (*n* = 25).

HER-2, human epidermal growth factor receptor 2; Neg, negative; Pos, positive; HIV, human immunodeficiency virus.

**TABLE 4:** KI-67 protein receptor status by human immunodeficiency virus status (*p* = 0.0383).

KI-67		HIV				Total
Variable	*p*	Neg		Pos		
		*n*	%	*n*	%	
Neg	<15%	32	53.33	12	30.77	44
Pos	>15%	28	46.67	27	69.23	55

**Total**	**-**	**60**	**100**	**39**	**100**	**99**

Note: Frequency missing (*n* = 30).

KI-67, KI-67 protein receptor; Neg, negative; Pos, positive; HIV, human immunodeficiency virus.

Molecular subtyping was possible in 96 of the patients with known HIV statuses. According to the criteria of the St Gallen 2013 Consensus Panel majority opinion,^22^ 19 patients possessed triple-negative breast cancer, of which 7 of 38 (18.42%) were HIV-positive and 12 of 58 (20.69%) were HIV-negative (*p* = 1.00). Around 55.26% of the HIV-positive patients and 46.55% of the HIV-negative patients in this group had Luminal B type cancers (*p* = 0.5315). HER2-enriched subtype was present in 13.16% of HIV-positive patients and 10.34% of the HIV-negative patients (*p* = 0.7483). None of these differences were statistically significant (Table 5).

**TABLE 5:** Molecular subtype by human immunodeficiency virus status.

Types	Subtypes	HIV					
		Neg		Pos		Total	
		*n*	%	*n*	%	*n*	%
Luminal A	-	13	22.41	5	13.17	18	-
Luminal B	-	27	46.55	21	55.26	48	-
	Luminal B HER-2 neg	15	-	11	-	26	-
	Luminal B HER-2 pos	11	-	9	-	20	-
	Luminal, HER-2 N/A, KI-67 > 14%	1	-	1	-	2	-
Luminal, HER2 N/A, KI-67 < 14%	-	0	-	0	-	0	-
HER2 enriched	-	6	10.34	5	13.16	11	--
Triple Neg	-	12	20.69	7	18.42	19	-

**Total**	**-**	**58**	**100**	**38**	**100**	**96**	**-**

HER-2, Human epidermal growth factor receptor 2; Neg, negative; Pos, positive; KI-67, KI-67 protein receptor; HIV, human immunodeficiency virus.

## Discussion

Since breast cancer is the most prevalent type of NADC among HIV-positive women,^7^ the relationship between HIV and breast cancer has gained great interest over the past decade, with multiple studies being published on the topic both locally and internationally.^5,6,7,13,14,18,19^ This study attempted to either strengthen or question the available data.

Both local and international studies have investigated the interaction between HIV and breast cancer, though only proved that patients living with HIV tend to present with breast cancer at a younger age, compared to their HIV-negative counterparts.^6,18^ Cubasch et al. observed similarities in the age distribution of HIV in the general population and in HIV patients with breast cancer in Soweto.^6^ In line with previous publications,^6,18,23^ the most striking finding of our study concerns the difference in age at presentation with breast cancer, which varied based on patient HIV status: the mean age of breast cancer presentation in the HIV-positive group (44.86 ± 9.00 years) was approximately 10 years younger than that of the HIV-negative group (53.18 ± 12.48 years), and of the general population (54.56 ± 13.62 years) (*p* < 0.0001) (Figure 3). This raises the question whether HIV-positive patients should be screened for breast cancer at a younger age than the general population.

In the present study, the stage at presentation was not significantly influenced by HIV status, which correlates with available data from preceding studies.^6,7,18,19^ Studies performed in the US^18,19^ reported similar stages at presentation for HIV-positive and HIV-negative patients. The patients tended to present at earlier stages compared to that of sub-Saharan Africa.^18^ Cubasch et al.^24^ found that in excess of 50% of their breast cancer patients presented with an advanced stage of disease, either stage 3 or 4, regardless of HIV status. It was then suggested that breast cancer may be detected at earlier stages in HIV-positive women if breast examination is included as standard of care at clinics providing primary health care to these women. Targeted education on breast self-examination may also be included.^24^

Age and stage at breast cancer presentation were found to be independent of the CD4 count of HIV-positive patients in Africa and sub-Saharan Africa.^7,11^ Moreover, international studies also failed to demonstrate a relationship between these variables.^18^ We, however, determined that as the CD4 count of HIV-positive patients decreased, the likelihood of presenting with advanced stages of breast cancer increased. The single patient with a CD4 count over 1000 cells/µL presented with stage 4 disease, which did not follow the trend demonstrated in the other groups (Figure 6). These results may be biased because of the small sample size and missing CD4 results for 10 (25.64%) of the patients. The CD4 count at the time of breast cancer diagnosis may affect chemotherapy tolerance; however, there is a paucity of available data.^11^ This warrants further investigation.

Similarly, no statistically significant differences were demonstrated for histological and molecular subtypes.^6,7,18,19^ The most common histological subtype, regardless of HIV status, was by far IDC – as expected. The lymphoproliferative neoplasm was observed in only one HIV-positive patient. When comparing the molecular subtypes, one must consider that results for the receptor status testing were not available for all the samples. This may have led to bias in the results.

## Conclusion

While HAART prolongs the survival of HIV-positive patients in South Africa, the incidence of NADCs is on the rise. An increasing number of patients – predominantly women – are facing both HIV and breast cancer. This study, albeit small, adds to the mounting evidence of breast cancer presenting earlier in women infected by HIV.

Because of the immense HIV burden in South Africa, it would be of great value to investigate its relationship with breast cancer on a national level. If the trends revealed in the present study (and others) are reproducible and generalisable to the South African population, it warrants a review of the management protocols for HIV-positive women.

Earlier screening for breast cancer in HIV-positive patients may decrease morbidity and mortality. Further research should be aimed at appropriate radiological screening methods for the younger population, while considering risk vs. benefit ratios, costs and the availability and diagnostic value of mammography, sonography and MRI. Furthermore, health care workers should be educated regarding the difference in breast cancer presentation in HIV-positive women, in order to increase their index of suspicion and ensure appropriate referral for further investigation and management. Patient knowledge and breast awareness should also be addressed by targeted education programmes.

## Study limitations

A major drawback of this study is that the HIV statuses of 51% of the small cohort of patients with breast cancer were unknown, which precludes calculation and comparison of HIV prevalence. Incomplete or missing histological results further limited the study. The presence of additional breast cancer risk factors was unaccounted for.
